# Rapid Perturbation in Viremia Levels Drives Increases in Functional Avidity of HIV-specific CD8 T Cells

**DOI:** 10.1371/journal.ppat.1003423

**Published:** 2013-07-04

**Authors:** Selena Viganò, Felicitas Bellutti Enders, Isabelle Miconnet, Cristina Cellerai, Anne-Laure Savoye, Virginie Rozot, Matthieu Perreau, Mohamed Faouzi, Khalid Ohmiti, Matthias Cavassini, Pierre-Alexandre Bart, Giuseppe Pantaleo, Alexandre Harari

**Affiliations:** 1 Service of Immunology and Allergy, Department of Medicine, Lausanne University Hospital, Lausanne, Switzerland; 2 The Center of Clinical Epidemiology, Institut de Médecine Sociale et Préventive, Lausanne University Hospital, Lausanne, Switzerland; 3 Service of Infectious Diseases, Department of Medicine, Lausanne University Hospital, Lausanne, Switzerland; 4 Swiss Vaccine Research Institute, Lausanne, Switzerland; Emory University, United States of America

## Abstract

The factors determining the functional avidity and its relationship with the broad heterogeneity of antiviral T cell responses remain partially understood. We investigated HIV-specific CD8 T cell responses in 85 patients with primary HIV infection (PHI) or chronic (progressive and non-progressive) infection. The functional avidity of HIV-specific CD8 T cells was not different between patients with progressive and non-progressive chronic infection. However, it was significantly lower in PHI patients at the time of diagnosis of acute infection and after control of virus replication following one year of successful antiretroviral therapy. High-avidity HIV-specific CD8 T cells expressed lower levels of CD27 and CD28 and were enriched in cells with an exhausted phenotype, *i.e.* co-expressing PD-1/2B4/CD160. Of note, a significant increase in the functional avidity of HIV-specific CD8 T cells occurred in early-treated PHI patients experiencing a virus rebound after spontaneous treatment interruption. This increase in functional avidity was associated with the accumulation of PD-1/2B4/CD160 positive cells, loss of polyfunctionality and increased TCR renewal. The increased TCR renewal may provide the mechanistic basis for the generation of high-avidity HIV-specific CD8 T cells. These results provide insights on the relationships between functional avidity, viremia, T-cell exhaustion and TCR renewal of antiviral CD8 T cell responses.

## Author Summary

CD8 T cells directed against virus are complex and functionally heterogeneous. One relevant component of CD8 T cells is their functional avidity which reflects their sensitivity to cognate antigens, *i.e.* how prone T cells are to respond when they encounter low doses of antigens. In patients with chronic and established HIV infection, we observed that the sensitivity of HIV-specific CD8 T cells was not different between patients with progressive or non-progressive disease. In contrast, the sensitivity of HIV-specific CD8 T cells was significantly lower in patients with early and recent HIV infection. Furthermore, CD8 T cells of high avidity were preferentially associated with a state of functional impairment known as exhaustion. Of interest, some patients treated with antiretroviral therapy during acute infection spontaneously interrupted their treatment and experienced a rebound of virus. In these patients, the avidity of HIV-specific CD8 T cells increased and this increase was associated to stronger cell exhaustion and greater renewal of the population of antiviral CD8 T cells, thus potentially providing the mechanistic basis for the generation of high-avidity CD8 T cells. Overall, our data suggest that rapid perturbation in viremia levels drove increases in the functional avidity of HIV-specific CD8 T cells.

## Introduction

CD8 T cells play a critical role in antiviral immunity and a large number of studies in both human and murine models indicate that virus-specific CD8 T cells are directly involved in the control of virus replication and disease progression [1], [2], [3], [4], [5], [6], [7].

Functional avidity of T cells, also defined as antigen (Ag) sensitivity, is thought to be a critical component of antiviral immunity. Functional avidity reflects the ability of T cells to respond to a low Ag dose and is determined by the threshold of Ag responsiveness. There is a general consensus that high functional avidity CD8 T-cell responses are of higher efficacy against cancers [8] and acute virus infections [9]. However, their relevance in chronic persistent virus infections and established tumors [10], [11], [12] remains to be determined since conflicting results were obtained in these contexts [13], [14] as well as in HIV infection [15], [16], [17], [18], [19]. HIV-specific CD8 T-cell responses in non-progressive infection were associated with high avidity and superior variants recognition [11], [12], [20], [21], whereas other studies indicated similar avidity between patients with progressive and non-progressive chronic infection [16], [18], [19], [22], [23]. In this regard, we have previously shown that polyfunctional virus-specific CD8 T-cell responses during chronic virus infections were predominantly of low functional avidity [24]. Furthermore, it is also well established that high functional avidity T-cell responses preferentially led to viral escape and T-cell clonal exhaustion [10], [24], [25], [26].

However, the factors determining the level of T-cell functional avidity and its relationship with the phenotypic and functional heterogeneity of T-cell responses are only partially understood [15], [16], [17], [18], [19], [22].

Functional avidity is based on the ability of T cells to respond following stimulation with a cognate Ag and it is well established that responding CD8 T cells are clonally heterogeneous (*i.e.* oligoclonal) [27], [28], [29], [30]. Therefore, the clonotypic composition of the responding T-cell population (and its TCR diversity) can influence functional avidity [27], [28]. Indeed, we and others reported that HIV-specific CD8 T cells responding to various epitopes harbor a diverse TCR repertoire in chronically-infected patients [31], [32], [33].

HIV-specific CD8 T cells in primary HIV infection (PHI) are temporally associated with the initial control of viremia [1]. Lichterfeld and colleagues suggested that high-avidity HIV-specific CD8 T-cell responses are present during early infection (defined as HIV seroconversion within 6 months) and are then preferentially lost overtime [33].

In the present study, we have performed a comprehensive cross-sectional characterization of HIV-specific CD8 T-cell responses in patients with PHI or chronic (progressive and non-progressive) HIV infection in both steady-state conditions as well as following virus rebound. The primary observations of the present study indicate that a) the functional avidity of HIV-specific CD8 T cells is not different between patients with progressive and non-progressive chronic infection, b) the functional avidity of HIV-specific CD8 T cells is significantly lower in PHI patients as compared to patients with chronic infections, c) increased functional avidity is associated with T-cell exhaustion and lack of expression of markers of co-stimulation, and d) great increase in functional avidity is observed after virus rebound following spontaneous interruption of antiretroviral therapy and is associated with increased TCR renewal.

## Results

### Lower functional avidity of HIV-specific CD8 T cell responses during acute infection

**Figure 1:**
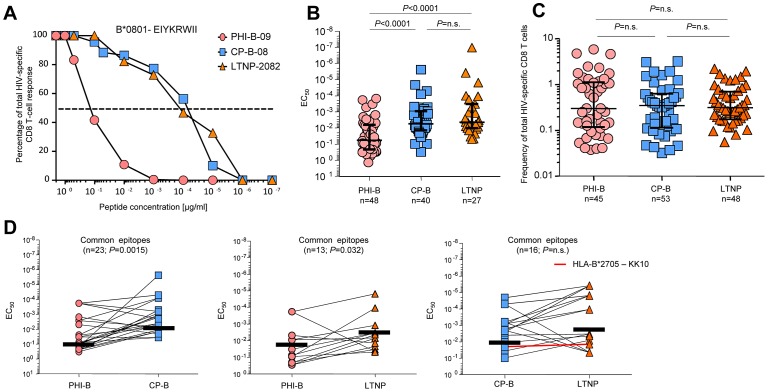
Functional avidity of HIV-specific CD8 T-cell responses. **A.** Representative examples of functional avidity of HIV-specific CD8 T-cell responses in patients with acute (PHI-B-09; circles), chronic progressive (CP-B-08; squares) or chronic non-progressive (LTNP-2082; triangles) infection after stimulation with decreasing concentrations of B*0801-_EIYKRWII_ peptides. The dashed line corresponds to half of the maximal response allowing the extrapolation of the 50% effect concentration (EC_50_). **B.** Cumulative analysis of the functional avidity of HIV-specific CD8 T cells during acute (PHI-B), untreated chronic progressive (CP-B) and non-progressive infection (LTNP). Medians and interquartile ranges are shown and each point represents one HIV-specific CD8 T-cell response. **C.** Cumulative analysis of the frequency of HIV-specific CD8 T cells during acute (PHI-B), untreated chronic progressive (CP-B) and non-progressive infection (LTNP). **D.** Functional avidity of HIV-specific CD8 T-cell responses against common optimal epitopes recognized by patients with acute (PHI-B), chronic progressive (CP-B) or not progressive (LTNP) infections. The red line (right panel) shows the functional avidity of B*2705-_KRWIILGLNK_ (KK10)-specific CD8 T-cell response.

We recruited 85 HIV-infected patients and performed a cross-sectional analysis of the functional avidity of HIV-specific CD8 T-cell responses. The distinct groups included a) 37 patients with very early stage of acute infection (*i.e.* prior to seroconversion and incomplete western blot; hereafter referred to as PHI), 39 patients with progressive chronic infection (*i.e.* typical progressors; hereafter referred to as CP) and 9 patients with non-progressive chronic infection (*i.e.* LTNP) (Table S1). We first investigated the 115 HIV-specific CD8 T-cell responses obtained in 26 untreated PHI (PHI-B), 19 untreated CP (CP-B) patients and 9 LTNP (Fig. 1A–B). As described in the Methods, blood mononuclear cells were stimulated with decreasing concentrations of the cognate peptides and the peptide dose able to induce half of the maximal response (*i.e*. effect concentration 50%; EC_50_) was determined (Fig. 1A).

The results of this analysis indicated that the functional avidity of HIV-specific CD8 T cells was lower in PHI-B as compared to CP-B or LTNP patients (both *P*<0.0001; Fig. 1A–B) while there was no difference between CP-B and LTNP (Fig. 1A–B). However, there were no significant difference in the magnitudes of HIV-specific CD8 T-cell responses among all groups (Fig. 1C) and no significant association between the functional avidity and the magnitude of HIV-specific CD8 T-cell responses (Fig. S1A). Furthermore, the differences in functional avidity of HIV-specific CD8 T cells between the different cohorts were not influenced by distinct peptides/MHC class I associations, since these differences remained significant when common epitopes (*i.e.* epitopes recognized by patients from distinct cohorts) were analyzed (Fig. 1D). Of note, the B*2705-_KRWIILGLNK_ (*i.e.* KK10) epitope has been previously reported as a protective epitope [28], [34], [35] and it was one of the common epitopes recognized by CP-B and LTNP patients. While the functional avidity of B*2705-KK10-specific CD8 T-cell responses in CP-B and LTNP patients was almost identical, it was rather low as compared to the other HIV-specific CD8 T-cell responses from both groups (Fig. 1D).

Taken together, these results indicate a lack of association between the functional avidity of HIV-specific CD8 T cells and virus control consistently with the recent study from Chen and colleagues [22]. Furthermore, the HIV-specific CD8 T-cell responses in acute infection have lower functional avidity than in chronic infection.

### Characterization of HIV-specific CD8 T cell responses disappearing after acute infection

**Figure 2:**
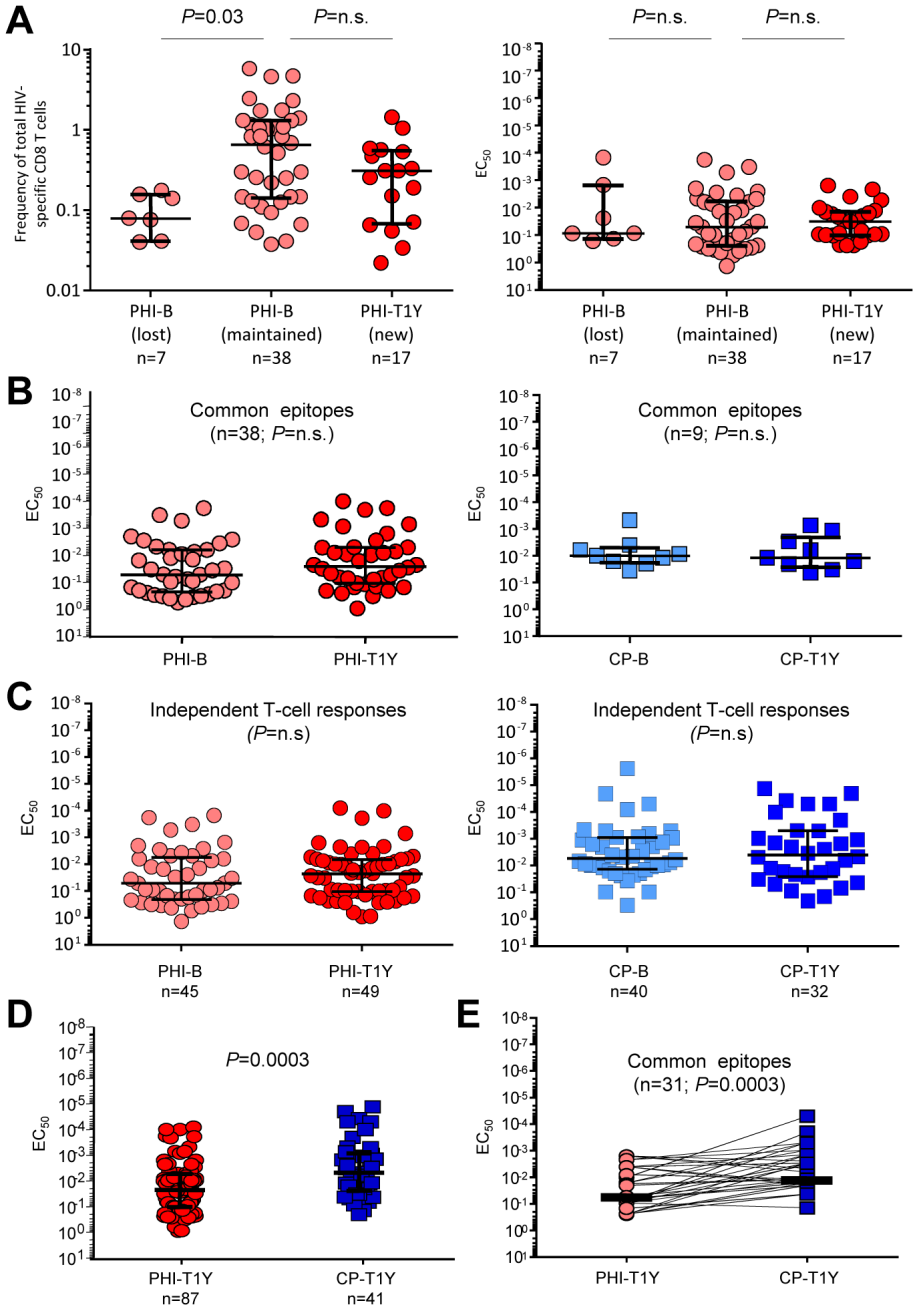
Analysis of the functional avidity of HIV-specific CD8 T-cell responses after one year of ART. **A.** Comparison of the magnitude (left panel) and functional avidity (right panel) of HIV-specific CD8 T-cell responses measured during acute infection (PHI-B) between responses which remained detectable (referred to as “maintained”) and those which became undetectable (referred to as “lost”) after one year of ART. Seventeen HIV-specific CD8 T-cell responses which were absent at baseline and developed during ART are also shown. **B.** Longitudinal matched-paired analysis of the functional avidity of HIV-specific CD8 T-cell responses measured before (-B) or after (-T1Y) 1 year of ART in patients with acute (PHI) or chronic progressive (CP) HIV infection. **C.** Functional avidity of independent HIV-specific CD8 T-cell responses detected in untreated (-B) or treated (-T1Y) patients with acute (PHI) or chronic progressive (CP) HIV infection. **D.** Functional avidity of HIV-specific CD8 T cells from patients with acute (PHI) or chronic progressive (CP) both after one year of ART (T1Y). **E.** Functional avidity of HIV-specific CD8 T-cell responses against common optimal epitopes recognized either by patients treated for 1 year since acute infection (PHI-T1Y) or treated for 1 year since chronic progressive infection (CP-T1Y).

It has been previously reported that high functional avidity HIV-specific CD8 T-cell responses are selectively deleted early after acute HIV infection [33]. We addressed this issue by repeating the epitopes mapping in patients with acute infection after one year of antiretroviral therapy (ART) (PHI-T1Y). Forty-five HIV-specific CD8 T-cell responses were identified in PHI-B patients using ICS. Among these 45 responses, 38 (85%) remained detectable after one year of ART whereas 7 (15%) became undetectable. Interestingly, at the time of acute infection, these 7 responses were already of lower magnitude as compared to the 38 responses which remained detectable (*P* = 0.03; Fig. 2A). Furthermore, the functional avidity of HIV-specific CD8 T-cell responses at the time of acute infection was not different between the 7 lost and the 38 remaining responses (*P*>0.05; Fig. 2A).

These results indicate that the minor proportion of HIV-specific CD8 T-cell responses selectively lost after acute infection did not have higher functional avidity.

### Functional avidity of HIV-specific CD8 T cell responses after one year of ART

It has been suggested that Ag load may influence the responsiveness of HIV-specific CD8 T cells [36]. To address this issue, we assessed whether the functional avidity of HIV-specific CD8 T-cell responses would change after control of virus replication, *i.e.* after 1 year of successful ART, in patients with acute or chronic infection. Of note, 46 additional HIV-specific CD8 T-cell responses were considered; 17 responses were identified in the initial 26 PHI patients following re-mapping after 1 year of ART and 29 responses were identified in 11 additional PHI patients only mapped after 1 year of ART. Both magnitude and functional avidity of HIV-specific CD8 T-cell responses generated during ART were similar to those measured at baseline (Fig. 2A). Furthermore, PHI patients were treated either with ART alone or with ART+CyclosporinA (CsA) but CSA treatment had no significant impact on the magnitude or the functional avidity of HIV-specific CD8 T-cell responses (Fig. S2).

The functional avidity of the same HIV-specific CD8 T-cell responses measured longitudinally either prior to ART or after 1 year of ART remained stable in both PHI and CP patients (both *P*>0.05; Fig. 2B). Furthermore, the lack of significant effect of ART on the functional avidity of HIV-specific CD8 T cells was also confirmed in non-longitudinal, independent, T-cell responses from PHI or CP patients (both *P*>0.05; Fig. 2C). Therefore, HIV-specific CD8 T-cell responses remained of lower avidity (*P* = 0.0003) in PHI-T1Y as compared to CP-T1Y patients (Fig. 2D). Consistently with the above-mentioned analyses performed in the untreated groups, differences in functional avidity of HIV-specific CD8 T cells between PHI-T1Y and CP-T1Y were not related to distinct peptide-MHC class I associations since the differences remained significant also when common epitopes were considered (*P* = 0.0003; Fig. 2E).

All together, these observations indicate that even after control of virus replication HIV-specific CD8 T-cell responses from patients with acute HIV infection remain of lower avidity as compared to patients with chronic infection.

### Functional and phenotypic profiles of HIV-specific CD8 T cells during acute and chronic HIV infections

We then assessed the functional profile of HIV-specific CD8 T cells from PHI-B, CP-B and LTNP patients. Although, the magnitudes of HIV-specific CD8 T-cell responses were not significantly different between PHI-B, CP-B and LTNP (Fig. 1C), perforin expression was significantly (*P*≤0.001) higher in HIV-specific CD8 T cells from PHI-B patients as compared to CP-B or LTNP (Fig. S1B and 3A). As previously shown [37], [38], HIV-specific CD8 T cells from LTNP contained more IL-2, whereas those from CP-B patients were mostly composed of single IFN-γ-producing cells (both *P*<0.0001; Fig. S1B and 3A).

**Figure 3:**
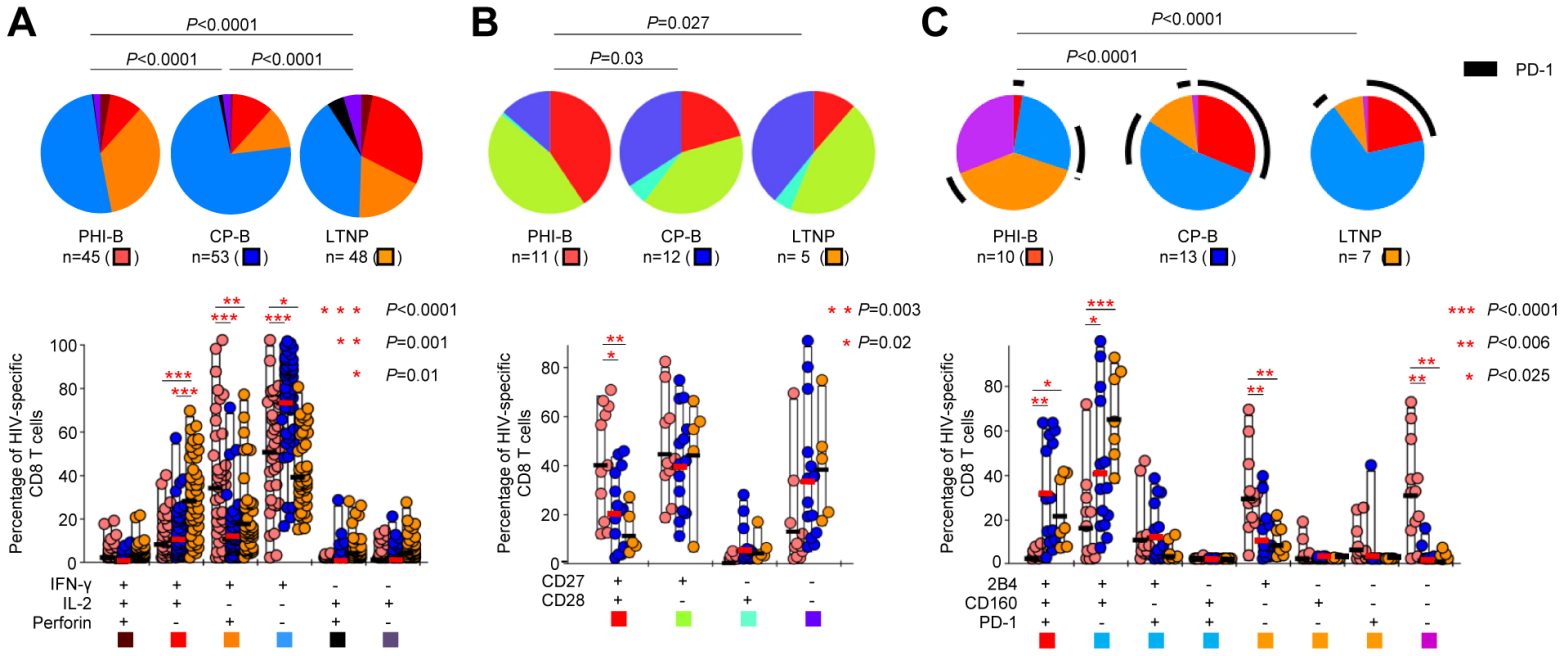
Functional profile and expression of co-stimulatory molecules and of co-inhibitory receptors of HIV-specific CD8 T-cell responses during acute and chronic HIV infections. Analysis of the functional profile (**A**), of the expression of CD27 and CD28 (**B**) and of the expression of 2B4, CD160 and PD-1 (**C**) in HIV-specific CD8 T cells in patients with acute (PHI-B), untreated chronic progressive (CP-B) or non-progressive (LTNP) HIV infection. Representative examples of the distinct flow cytometry panels are shown in Fig. S1B-D. Regarding the functional profile (**A**), although TNF-α was detected, analyses are restricted to the expression of IFN-γ, IL-2 and perforin for clarity. All possible combinations of the distinct markers are shown on the *x* axis, whereas the percentages of the distinct cell subsets within virus-specific CD8 T cells are shown on the *y* axis. The pie charts summarize the data, and each slice corresponds to a certain combination of molecules. Colors in the pie charts are based on the colored boxes at the bottom of the panel.

We then performed a phenotypic characterization of HIV-specific CD8 T-cell responses and monitored CD27 and CD28 expression to assess co-stimulation and PD-1, 2B4 and CD160 expression to assess T-cell activation and exhaustion. For these analyses, only HIV-specific CD8 T cells detectable using cognate peptide-MHC class I multimers (Table S2) were taken into consideration. Regarding T-cell co-stimulation, HIV-specific CD8 T cells from PHI-B expressed a higher proportion of CD27^+^CD28^+^ cells than those from CP-B (*P* = 0.02) or LTNP (*P* = 0.003) patients (Fig. S1C and 3B). Also, analyses of the expression of co-inhibitory receptors indicated that HIV-specific CD8 T cells from CP-B and LTNP were both composed of significantly higher proportions of PD-1^+^2B4^+^CD160^+^ (*P*<0.006 and *P*<0.025, respectively) or PD-1^−^2B4^+^CD160^+^ (*P*<0.025 and *P*<0.0001, respectively) as compared to PHI-B (Fig. S1D and 3C). HIV-specific CD8 T cells from PHI-B mostly (about 70%) lacked all three markers or expressed 2B4 alone (all *P*<0.006; Fig. 3C) and expressed lower frequency and intensity of PD-1 as compared to CP-B (both *P*<0.002; data not shown).

These data suggest that HIV-specific CD8 T cells from patients with acute and chronic infection are functionally and phenotypically distinct.

### Association between the functional avidity and the phenotypic and functional heterogeneity of HIV-specific CD8 T cells

**Figure 4:**
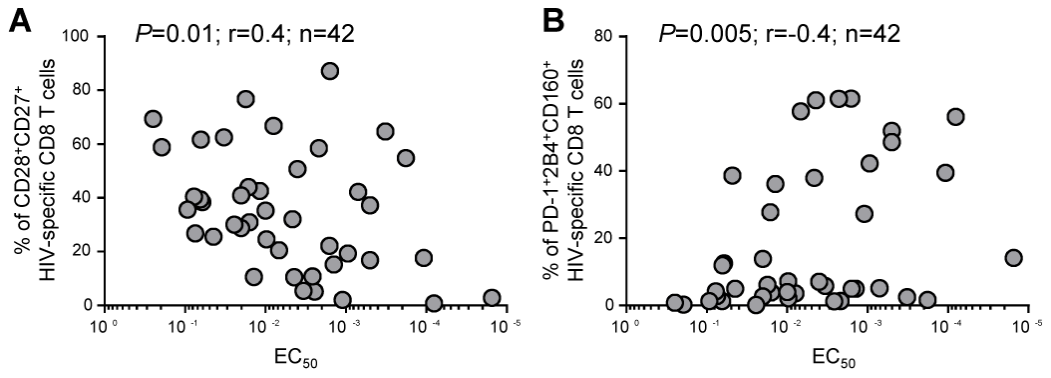
Associations between functional avidity of HIV-specific CD8 T-cell responses and other immunological parameters. Associations between the functional avidity of HIV-specific CD8 T-cell responses and the frequency of HIV-specific CD8 T cells co-expressing CD28 and CD27 (**A**) or PD-1, 2B4 and CD160 (**B**).

We then assessed the association between functional avidity and the expression of co-stimulatory or co-inhibitory receptors. The functional avidity of HIV-specific CD8 T cells was negatively correlated to the proportion of CD27^+^CD28^+^ cells (*P* = 0.01; Fig. 4A) and directly correlated to the proportion of cells co-expressing PD-1/2B4/CD160 (*P* = 0.005; Fig. 4B).

Furthermore, we performed correlations and rank correlation's matrix to explore the partial associations of variables and to assess the dependency and potential hidden effect of confounding variables in pairs associations. These analyses indicated that the proportions of CD27^+^CD28^+^ and of PD-1^+^2B4^+^CD160^+^ HIV-specific CD8 T cells were not significantly dependent on each other. This allowed us to perform a regression model analysis and to postulate that the functional avidity of HIV-specific CD8 T cells may be a linear function of the two aforementioned explained variables (after log_10_ transformation). Interestingly, this regression analysis indicated that about 28% of the functional avidity of HIV-specific CD8 T cells was explained by a combination of the proportion of CD27^+^CD28^+^ and of PD-1^+^2B4^+^CD160^+^ CD8 T cells (*P* = 0.0013; data not shown). We did not, in contrast, observe any significant correlation between the functional avidity of HIV-specific CD8 T cells and their functional profile.

Overall, these observations indicate that high-avidity HIV-specific CD8 T-cell responses are preferentially composed of cells lacking the expression of co-stimulatory molecules but co-expressing high levels of co-inhibitory receptors. However, the functional avidity can only be partially predicted from the expression of co-stimulatory or co-inhibitory molecules.

### Qualitative changes of HIV-specific CD8 T cells in patients experiencing a virus rebound following treatment interruption

**Figure 5:**
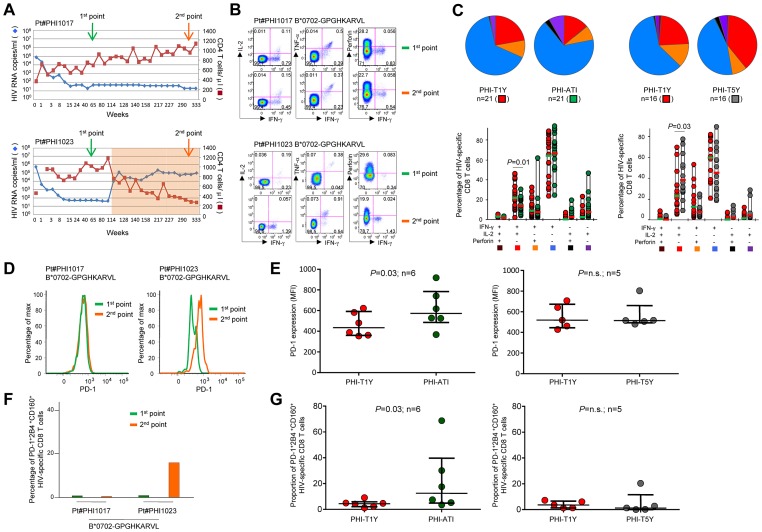
Qualitative changes of HIV-specific CD8 T cells in patients experiencing a virus rebound following treatment interruption. **A.** Longitudinal analysis of the CD4 T-cell counts and HIV viremia of 2 representative HIV-infected patients (#1017 and #1023) treated with ART since acute infection. Patient #1017 remained on ART, whereas patient #1023 spontaneously interrupted ART after 119 weeks (orange box). **B.** Functional profiles of B*0702-_GPGHKARVL_-specific CD8 T cells from patients #1017 and #1023 analyzed at two distinct time-points corresponding to weeks 48 and 300 (identified by the arrows in A). **C.** Cumulative analysis of the functional profile of HIV-specific CD8 T-cell responses on the basis of the expression of IFN-γ, IL-2 and perforin in patients with acute (PHI) infection after one year of ART (-T1Y) and after either treatment interruption (-ATI) or after five years of ART (-T5Y). Matched-paired HIV-specific CD8 T-cell responses are considered for the comparison between T1Y and ATI or between T1Y and T5Y. Although TNF-α was also detected, analysis is restricted to the expression of IFN-γ, IL-2 and perforin for clarity. All possible combinations of IFN-γ, IL-2 and perforin are shown on the *x* axis, whereas the percentages of the distinct cell subsets within HIV-specific CD8 T cells are shown on the *y* axis. The pie charts summarize the data, and each slice corresponds to a certain combination of functions. Colors in the pie charts are based on the colored boxes at the bottom of the panel. **D.** PD-1 expression in B*0702-_GPGHKARVL_-specific CD8 T cells in patients #1017 and #1023 measured at the two time-points identified with arrows in A. **E.** Mean fluorescence intensity (MFI) of PD-1 expression in HIV-specific CD8 T cells measured in patients with acute (PHI) HIV infection after 1 year of ART (-T1Y) and after either treatment interruption (-ATI, left panel) or after five years of ART (-T5Y, right panel). **F.** Proportion of PD-1^+^2B4^+^CD160^+^ cells in B*0702-_GPGHKARVL_-specific CD8 T cells in patients #1017 and #1023 measured at the two time-points identified with arrows in A. **G.** Proportion of PD-1^+^2B4^+^CD160^+^ cells in HIV-specific CD8 T cells measured in patients with acute (PHI) HIV infection after 1 year of ART (-T1Y) and after either treatment interruption (-ATI, left panel) or after five years of ART (-T5Y, right panel).

We then performed a longitudinal analysis to investigate the effect of changes in viremia levels on HIV-specific CD8 T cells. To address this issue, we longitudinally monitored HIV-specific CD8 T cells in two distinct models: a) in conditions of viremia below the limit of detection, *i.e.* viremia <50 HIV RNA copies/ml of plasma (in patients successfully treated by ART) and b) in conditions of rapid and major changes in viremia occurring in patients experiencing virus rebound following spontaneous treatment interruption (TI) (Fig. 5A). In particular, we evaluated HIV-specific CD8 T-cell responses in PHI-T1Y and compared them with those after 5 years (PHI-T5Y) of uninterrupted successful ART or after TI (PHI-ATI) (Fig. 5A). Nine out of the 37 patients identified during acute infection spontaneously interrupted ART. These patients were treated since PHI for ≥1 year (mean±SE 131±15 weeks) and all had undetectable viremia (<50 HIV RNA copies/ml) at the time of TI. After TI, all patients experienced a virus rebound with an average plasma viremia of 5.18 log_10_ HIV RNA copies/ml.

The functional profile of HIV-specific CD8 T-cell responses at the time of TI was different from that of baseline. HIV-specific CD8 T cells in PHI-T1Y were mostly polyfunctional (associated to a large fraction of IL-2-producing cells and little perforin) (*P*<0.0001; Fig. 5B–C) as compared to the typical effector profile (Fig. 3A) observed in PHI-B. In patients remaining on ART, HIV-specific CD8 T cells became more polyfunctional (*i.e.* further shifted toward IL-2 production) after 5 years of ART as compared to 1 year of ART (Fig. 5B–C). Conversely, in patients interrupting ART, as shown for patient #1023 who interrupted ART after two years of treatment and experienced a virus rebound of 122'000 HIV RNA copies/ml, the proportion of HIV-specific CD8 T cells co-producing IFN-γ and IL-2 decreased (Fig. 5A–B). Cumulative analyses confirmed the significant (*P*<0.01) decrease in polyfunctionality of HIV-specific CD8 T-cell responses after TI and the significant (*P* = 0.03) increase in polyfunctionality of HIV-specific CD8 T-cell responses from patients who remained on ART (Fig. 5C).

Then we determined PD-1 (as well as 2B4 and CD160) expression in a subset of PHI-ATI and PHI-T5Y patients with known HIV-specific CD8 T-cell responses using cognate peptide-MHC class I multimers (Table S2). As shown in the representative flow cytometry profiles from patients #1017 and #1023, PD-1 expression increased in patient #1023 who interrupted ART but not in patient #1017 who remained on ART (Fig. 5D). Along the same line, the proportion of triple PD-1^+^2B4^+^CD160^+^ HIV-specific CD8 T cells also increased in patient #1023 but not in patient #1017 (Fig. 5F). Cumulative analyses confirmed that PD-1 expression as well as the proportion of cells co-expressing PD-1/2B4/CD160 in HIV-specific CD8 T cells were significantly increased in patients who interrupted ART (both *P* = 0.03; Fig. 5E and 5G). No increase in PD-1 expression or in the co-expression of PD-1/2B4/CD160, however, was observed in the patients who remained on ART (both *P*>0.05; Fig. 5E and G). Finally, consistently with the differences in the functional profile of HIV-specific CD8 T-cell responses between patients who did or did not interrupt ART (Fig. 5B–C), we observed that the proportion of dual IFN-γ/IL-2-producing HIV-specific CD8 T cells was negatively correlated with the proportion of cells co-expressing PD-1/2B4/CD160 (*P* = 0.009; data not shown).

These data indicate that major changes of viremia levels in TI patients caused reduction of polyfunctional HIV-specific CD8 T cells and were associated with an increased level of exhaustion.

### Increase in functional avidity of HIV-specific CD8 T cells following TI

We then analyzed the effects of virus rebound following TI on the functional avidity of HIV-specific CD8 T cells.

**Figure 6:**
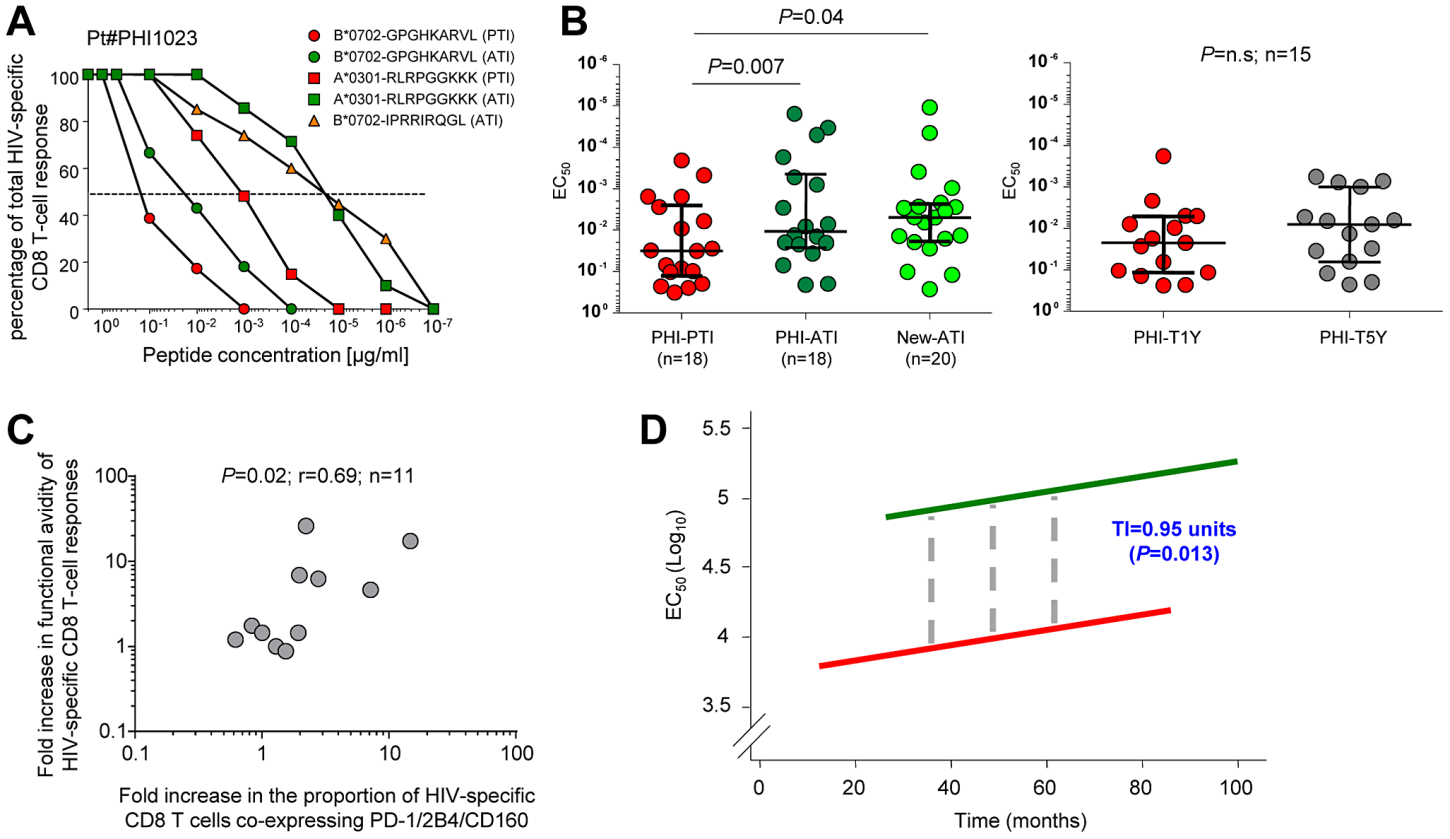
Increase in functional avidity of HIV-specific CD8 T cells following treatment interruption. **A.** Functional avidity of B*0702-_GPGHKARVL_- and A*0301-_RLRPGGKKK_-specific CD8 T cells from patient #1023 before (-PTI) and after (-ATI) treatment interruption (TI) and analysis of the functional avidity of B*0702-_IPRRIRQG_-specific CD8 T-cell response, which was undetectable before TI. **B.** Cumulative matched-paired analysis of the functional avidity of HIV-specific CD8 T-cell responses in patients treated during acute HIV infection (PHI) before (-PTI) or after treatment interruption (-ATI) and in PHI patients after 1 (-T1Y) or 5 years (-T5Y) of uninterrupted ART. Twenty new HIV-specific CD8 T-cell responses generated ATI (New-ATI) within the same patients are also shown. **C.** Association between the fold increase in avidity of HIV-specific CD8 T-cell responses and the fold increase in the proportion of HIV-specific CD8 T cells co-expressing PD-1, 2B4 and CD160. **D.** Mixed-effect linear models were used to assess the evolution of functional avidity as a function of time and virus rebound, as detailed in the Methods. Solid lines represent the lack of significant increase in avidity overtime in patient ON (red line) or OFF (green line) ART, whereas the dashed lines represent the immediate increase of functional avidity occurring after treatment interruption.

As shown for patient #1023, the functional avidity of B*0701-_GPGHKARVL_- and A*0301-_RLRPGGKKK_-specific CD8 T-cell responses significantly increased after TI (ATI) as compared to pre-TI (PTI) (Fig. 6A). Furthermore, an additional HIV-specific CD8 T-cell response against B*0701-_IPRRIRQGL_, which was below the detection level PTI, was observed ATI (Fig. 6A). Cumulative analyses confirmed the increase in functional avidity of HIV-specific CD8 T cells occurring ATI (*P* = 0.007; Fig. 6B) and also indicated that new responses generated following virus rebound were of high avidity (*P* = 0.04; Fig. 6B). Consistently, no significant (*P*>0.05) differences in functional avidity were observed during the same period when a similar analysis was performed in HIV-specific CD8 T-cell responses from patients who did not interrupt ART (*i.e.* for an average of 4 years; Fig. 6B). Furthermore, the functional avidity of HIV-specific CD8 T-cell responses ATI was in the same range as compared to those observed in CP patients (data not shown). Of note, consistently with the increase in the co-expression of co-inhibitory molecules occurring in patients experiencing virus rebound (Fig. 5F–G), we observed a positive association (*P* = 0.02) between the fold increase in functional avidity of HIV-specific CD8 T cells and the fold increase in the proportion of PD-1^+^2B4^+^CD160^+^ HIV-specific CD8 T cells (Fig. 6C).

Of interest, we performed a comprehensive statistical modeling of the changes in functional avidity of HIV-specific CD8 T cells and used mixed-effect linear models [39], [40] to assess the evolution of functional avidity as a function of time and virus rebound.

For this analysis, all longitudinal measures (n = 231) of functional avidity were included. The statistical model revealed that the interaction between avidity and time was not significant in steady-state conditions, *i.e.* neither in patients on ART (Fig. 6D; red line), nor in patients off ART (Fig. 6D; green line). In both conditions, an increase of 0.013 units per month was determined but did not reach statistical significance (*P* = 0.05; Fig. 6D), thus indicating that functional avidity does not significantly change under steady-state circumstances. However, we found a significant (*P* = 0.013) interaction between functional avidity and virus rebound. An immediate increase of functional avidity of HIV-specific CD8 T cells of about 1 order of magnitude (0.95 units) occurred directly after TI (Fig. 6D, grey dashed lines) and was not related to the duration of ART prior to TI.

These observations indicated that the functional avidity of HIV-specific CD8 T cells is stable overtime in steady-state conditions regardless of viremia levels, but does increase after rapid increase in viremia levels associated with virus rebound.

### Association between CDR3 renewal and increase in functional avidity of HIV-specific CD8 T cells

We recently demonstrated that the global CD8 TCR repertoire of virus-specific CD8 T cells was diverse and subjected to continuous renewal [32]. We then evaluated the TCR repertoire in PHI patients experiencing a virus rebound following TI. For this purpose, we measured CDR3 diversity and the percentage of renewal of HIV-specific CD8 T cells and compared those to the changes in functional avidity of HIV-specific CD8 T cells occurring before and after virus rebound.

**Figure 7:**
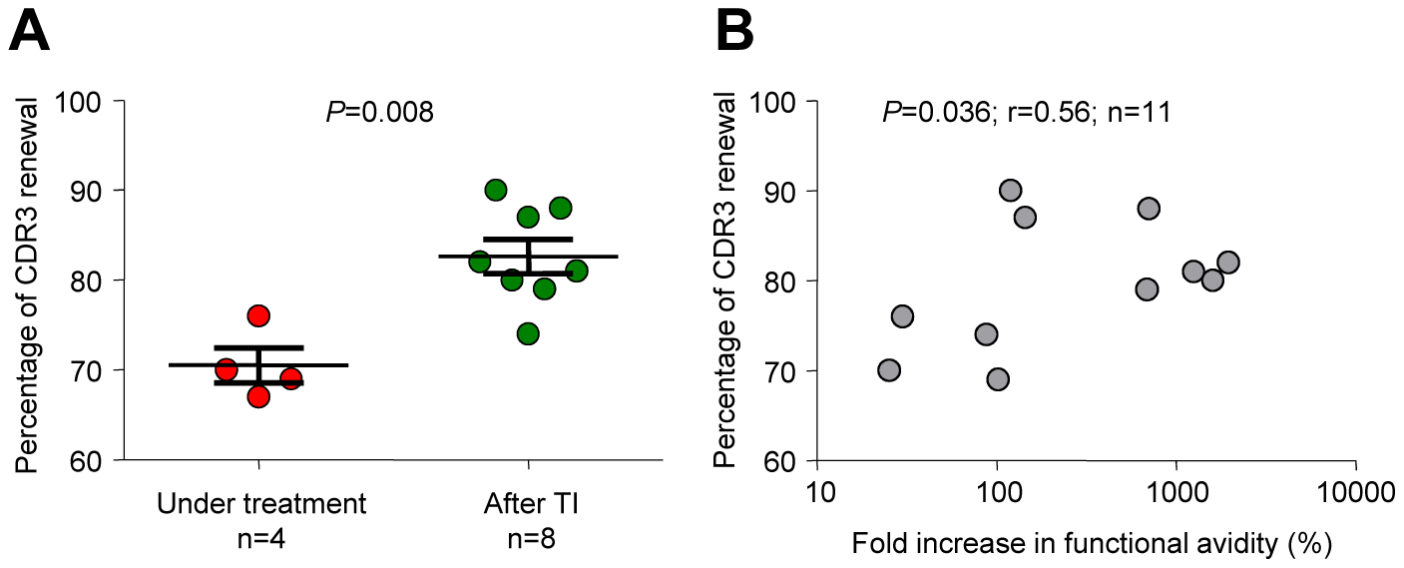
Increased CDR3 renewal of HIV-specific CD8 T cells following treatment interruption and association with functional avidity. **A.** Percentage of CDR3 renewal of HIV-specific CD8 T cells before (under treatment) and after treatment interruption (TI). CDR3 diversity and renewal were determined as described [32]. Example of TRBV usage and CDR3 size pattern analysis of B*0702-_GPGHKARVL_-specific CD8 T cells in patient #1023 at week 18, 96 and 125 are shown in Fig. S3. **B.** Association between the percentage of CDR3 renewal and changes in the functional avidity of HIV-specific CD8 T cells.

As shown for patient #1023 (Fig. 5A), TRBV usage and CDR3 size pattern were analyzed for B*0701-_GPGHKARVL_-specific CD8 T cells at week (W) 18, W96 and W125 (Fig. S3A–B). Using our previously-described model to determine CDR3 renewal [32], we calculated a renewal of 76% between W18 and W96 (*i.e.* on ART) and a renewal of 82% between W96 and W125 (*i.e.* after TI). Cumulative analyses confirmed a significantly (*P* = 0.008) higher CDR3 renewal of HIV-specific CD8 T cells after virus rebound than in steady-state condition, *i.e.* during treatment (Fig. 7A). Interestingly, the level of CDR3 renewal was directly associated (*P* = 0.036) with the extent of increase in functional avidity of HIV-specific CD8 T cells (Fig. 7B).

Taken together, these observations suggest that increase in CDR3 renewal may contribute to the increase in functional avidity of HIV-specific CD8 T cells occurring after virus rebound.

## Discussion

T-cell functional avidity reflects the ability of T cells to respond to various concentrations of Ag and may be assessed *ex vivo* through a quantification of a biological function such as IFN-γ production, cytotoxic activity or proliferation capacity. Several parameters concur to determine the threshold of T-cell responsiveness. These include: a) the affinity of the TCR for the peptide-MHC (pMHC) molecule, *i.e.* the strength of the interaction between the TCR and pMHC [41], [42], b) the density of pMHC-TCR interactions (reflecting both the amount of Ag and the ability of Ag presenting cells (APC) to present Ags) [43], [44], [45], [46], c) the expression of co-stimulatory and co-inhibitory molecules by T cells and APC [47], and d) the T-cell distribution and composition of signaling molecules [44], [48]. However, the factors determining functional avidity and the relationship between functional avidity and the heterogeneity of T-cell responses are not well understood.

In the present study, we comprehensively investigated the functional avidity of HIV-specific CD8 T-cell responses in a cross-sectional study of different cohorts of HIV-infected patients. The evaluation of the functional avidity of HIV-specific CD8 T cells was based on optimal epitopes, *i.e.* epitopes not necessarily corresponding to the autologous virus sequences. Since pMHC/TCR affinity is one of the parameters potentially influencing the functional avidity [49], [50], a mismatch between the epitope sequences and the TCR or the MHC may impact the determination of avidity. However, the same strategy was used throughout all cohorts of HIV-infected patients, thus minimizing the potential biases in our observations.

HIV-specific CD8 T cells generated during acute infection were of lower functional avidity as compared to those from patients with chronic progressive or non-progressive infection. These differences were not biased by distinct peptide-HLA associations and remained significant after ART-induced control of virus replication. In addition, a preferential deletion of HIV-specific CD8 T-cell responses of higher avidity was not observed, as previously described in a cohort of early HIV infection [33]. The discrepancy between our and the previous study may be explained by differences in the individual cohorts as well as by the fact that all the 37 patients received ART at the time of diagnosis of PHI in our study, whereas only 5 of the 10 patients in Lichterfeld's study received ART [33]. Our results also indicated that the minor proportion of HIV-specific CD8 T-cell responses lost after acute infection had an initial lower magnitude rather than a higher avidity.

Furthermore, consistently with previous studies [16], [18], [19], [22], [23], [51], there were no significant differences in the functional avidity of HIV-specific CD8 T-cell responses from chronic progressive and non-progressive infection. These observations suggest that T-cell functional avidity does not represent a correlate of virus control, at least in the context of chronic and persistent virus infections. Along the same line, HIV-specific CD8 T-cell responses commonly associated with virus control [52], *i.e.* HLA-B*27-, B*57- or B*5801-restricted T-cell responses, were consistently found in the lower range of functional avidity (data not shown). These observations do not support previous studies showing a relationship between higher avidity T-cell responses and better virus control [11], [12], [20], [21], [53], [54]. One potential explanation is that in most of these studies, specific T-cell epitopes (e.g. TW10 or KK10) were considered predominantly in individuals with non-progressive infection. Of note, consistently with our study, Chen and colleagues recently demonstrated that KK10-specific CD8 T-cell responses in elite controllers showed better virus control and broader viral recognition but similar functional avidity as compared to progressors [22]. They also confirmed the overall lack of difference in the functional avidity of HIV-specific CD8 T cells between patients with progressive and non-progressive infection [22].

Taken together, these observations suggest an association between higher avidity T-cell responses and chronic HIV infection.

Of note, we also assessed the relationship between T-cell functional avidity and the expression of markers of exhaustion. It is important to underscore that HIV-specific CD8 T cells which had higher avidity in chronic infection expressed also higher levels of exhaustion markers. Therefore, these results further indicate that higher functional avidity does not correlate with better virus control but rather with the status of cells activation/exhaustion.

We also determined the impact of the expression of costimulation (*i.e.* CD27 and CD28) and exhaustion markers (*i.e.* PD-1, CD160 and 2B4) on the levels of functional avidity in HIV-specific CD8 T cells using a regression model. The regression model indicated that the expression of the above markers only partially accounts for the establishment of the functional avidity of HIV-specific CD8 T cells, thus indicating that additional factors may contribute to determine the levels of functional avidity.

The lower avidity of HIV-specific CD8 T cells in PHI patients may also be potentially explained by the fact that patients were identified very early in the course of infection and received ART within 24 hours. Therefore, one cannot exclude the possibility that this early control of virus replication blunted the natural evolution and maturation of the immune response, as previously shown for T- and B-cell responses [55], [56], [57]. Consistently, it was also shown in mice that functional avidity of antiviral CD8 T cells continuously increased (avidity maturation) during the first month of infection [58].

In this regard, when patients treated during PHI experienced a virus rebound, the functional avidity of HIV-specific CD8 T-cell responses significantly increased. The mixed-effect linear model we used indicated a punctual increase of about one order of magnitude following virus rebound. However, there was no quantitative correlation between either the peak or the steady-state of the virus rebound and the increase in avidity.

Several mechanisms were proposed to modulate T-cell functional avidity maturation including: 1) the formation of clusters comprising several TCRs and other molecules able to reinforce the immunological synapses [59], [60], [61], 2) the optimization of the signal transduction machinery such as an increase in the amount of and in the basal phosphorylation levels of signaling molecules [62], [63] and 3) a selective expansion of high TCR avidity clones and/or the loss of clones with low TCR avidity [33], [64], [65], [66], [67]. We cannot exclude that the same mechanisms may also contribute to explain the increase in avidity observed following treatment interruption and virus rebound.

Interestingly, we showed that TCR renewal was also significantly higher following virus rebound and associated with an increase in T-cell functional avidity. Therefore, our data indicate a potential role of TCR renewal in the modulation of the levels of functional avidity. However, our results do not distinguish between the recruitment of new clones, selective expansion of pre-existing high-avidity clones or depletion of low-avidity clones since the study was performed at the population level.

Taken together, these results support the following model.

HIV-specific CD8 T cells of lower functional avidity are generated during primary immune responses; then, persistence of detectable viremia drives an increase in functional avidity as supported by the major increase in functional avidity associated with the sudden increment in viremia levels; the increase in viremia levels is also associated with massive TCR renewal which, in turn, causes the generation/selection of T-cell clones with higher functional avidity.

These results provide insights on the relationships between functional avidity, viremia, T-cell exhaustion and TCR renewal of antiviral CD8 T-cell responses.

## Methods

### Ethics statement

These studies were approved by the Institutional Review Board of the Centre Hospitalier Universitaire Vaudois and all subjects gave written informed consent.

### Study groups

Seventy-six patients with primary (PHI) or progressive chronic (CP) HIV infection were enrolled. Diagnosis of PHI included the presence of an acute clinical syndrome, a negative HIV antibody test, a positive test for HIV RNA in plasma, and ≤3 positive bands in a Western blot. All PHI patients started ART alone or ART+CsA within 72 h as described [68] and were followed for up to 10 years. Patients with chronic progressive (CP) HIV infection were infected for more than a year, were ART-naïve at the time of inclusion, had ≥400 CD4 T-cells/µl, ≥5000 plasma HIV RNA copies/ml and were directly treated with ART upon diagnosis as described [69], [70]. Four CP patients were investigated both prior to (BSL) and then after 1 year of ART (T1Y). Furthermore, 9 additional HIV-infected patients with non-progressive disease, *i.e.* LTNP, as defined by documented HIV infection since >10 years, stable CD4 T-cell counts >500 cells/µl, and plasma viremia <500 HIV RNA copies/ml were also included. Clinical and virological characteristics of the different cohorts are detailed in Table S1.

### Synthetic peptides and peptide-MHC class I multimer complexes

Epitope mapping was performed using a panel of 192 HPLC-purified (>80% purity) previously-described optimal epitopes [71]. Confirmation of specificity was achieved based on the HLA class I genotype of the patients and ICS assays. Peptide-MHC class I multimers (listed in Table S2) were purchased from ProImmune (Oxford, UK) except HLA-B*0801-RAKFKQLL, HLA-B*0702-RPPIFIRRL, HLA-B*0702-TPRVTGGGAM and HLA-B*5701-TSTLQEQIGW (Table S2) which were produced as described [72].

### Antibodies

The following antibodies were used in different combinations. CD8-PB, CD8-APCH7, CD3-APCH7, CD45RA-PECy5, PD-1-FITC, IFN-γ-APC, TNF-α-PECy7, and IL-2-PE were purchased from Becton Dickinson (BD, San Diego, CA), CD4-ECD, CD3-ECD, CD28-ECD, CD27-APC from Beckman Coulter (Fullerton, CA, USA), Perforin-FITC (clone B-D48) from Diaclone (Besançon, France), CCR7-FITC from R&D Systems (Minneapolis, MN, USA), 2B4-PECy5.5 and CD160-APC from Biolegend (San Diego, CA, USA).

### IFN-γ ELISpot assay

ELISPOT assays were performed *as per* the manufacturer's instructions (BD Biosciences). In brief, 2×10^5^ cryo-preserved blood mononuclear cells were stimulated with 1 µg of single peptide or peptide pools in triplicate conditions as described [73]. Media only and staphylococcal enterotoxin B (SEB) were used as negative and positive controls, respectively. Thresholds for assay validation and positivity were determined as described [73]. Results are expressed as the mean number of SFU/10^6^ cells from triplicate assays. Only cell samples with >80% viability after thawing were analyzed, and only assays with <50 spot forming unit (SFU)/10^6^ cells for the negative control and >500 SFU/10^6^ cells after SEB stimulation were considered valid. An ELISpot result was defined as positive if the number of SFUs was ≥55 SFU/10^6^ cells and ≥4-fold the negative control.

### *Ex vivo* analysis of virus-specific CD8 T cells

Cryo-preserved blood mononuclear cells (1–2×10^6^) were stained for dead cells (4°C for 20′; Aqua LIVE/DEAD, Invitrogen) and then stained with appropriately tittered peptide-MHC class I tetramer complexes at 4°C for 30′ in Ca^2+^-free media as described [74]. Cells were then washed and directly stained at 4°C for 20′ with the following Abs in various combinations: CD3, CD8, CD28, CD27, PD-1, 2B4, CD160. Finally, cells were fixed (CellFix, BD), acquired on an LSRII SORP (4 lasers) and analyzed using FlowJo 8.8.2 (Tree star Inc, USA). Analysis and presentation of distributions were performed using SPICE version 5.1, downloaded from http://exon.niaid.nih.gov/spice/ [75]. The number of lymphocyte-gated events ranged between 0.6–1×10^6^ in the flow cytometry experiments.

### ICS assay

Cryo-preserved blood mononuclear cells (1–2×10^6^) were stimulated for 6 h or overnight in 1 ml of complete media (RPMI (Invitrogen), 10% fetal bovine serum (FBS; Invitrogen), 100 µg/ml penicillin, 100 units/ml streptomycin (BioConcept)) in the presence of Golgiplug (1 µl/ml, BD), anti-CD28 (0.5 µg/ml, BD) and 1 µg/ml of peptide as described [74]. Staphylococcus enterotoxin B (SEB; Sigma) stimulation (100 ng/ml) served as positive control. At the end of the stimulation period, cells were stained for dead cells (4°C for 20′; Aqua LIVE/DEAD, Invitrogen), permeabilized (RT for 20′; Cytofix/Cytoperm, BD) and then stained at RT for 20′ with CD4, CD8, CD3, IFN-γ, IL-2, TNF-α and perforin (clone B-D48). Cells were then fixed (CellFix, BD), acquired on an LSRII SORP and analyzed using FlowJo 8.8.2. Analysis and presentation of distributions were performed using SPICE version 5.1, downloaded from http://exon.niaid.nih.gov/spice/ [75]. The number of lymphocyte-gated events ranged between 0.6–1×10^6^. With regard to the criteria of positivity of the ICS, the background in the unstimulated controls never exceeded 0.03%. An ICS to be considered positive had to have >0.03% of cytokine-positive cells after subtraction of the background (media alone) and to be >5 fold higher that the background.

### Determination of CDR3 renewal

The analysis of the CDR3 diversity and renewal was performed as described [32]. CDR3 renewal corresponds to the percentage of TCR sequences specific for a given epitope that changed between two time points. Briefly, blood mononuclear cells were stained with cognate multimers and anti-CD3, anti-CD8, anti-CD45RA, and anti-CCR7 mAbs (BD Biosciences). CD45RA^+^ CCR7^+^ naïve and Ag-specific (multimer^+^) CD8 T cells were directly sorted (FACSAria, BD Biosciences) in RLT lysis buffer (Qiagen, Hilden, Germany) containing 20 ng RNA carrier (Roche Diagnostics, Rotkreuz, Switzerland) and RNA extracted (Qiagen). Then, cDNA preparation and amplification were performed by using the SuperSMART PCR cDNA Synthesis Kit according to the manufacturer's instructions (Clontech Laboratories, Saint-Germain-en-Laye, France). Amplified cDNA was subjected to TRBV–TCR-b–chain C region (TRBC) PCR reactions as described [32]. For spectratyping, aliquots of positive samples were mixed with Genescan-500 ROX size standards and run on an ABI 3130 capillary sequencer (Applied Biosystems, Foster City, CA). The CDR3 junction (length) was analyzed using the IMGT system as described [32].

### Functional avidity

Peptide stimulations were performed as described above. Functional avidity of T-cell responses was assessed by performing limiting peptide dilutions (ranging from 2 µg/ml to 1 pg/ml) in *in vitro* assays as described [24]. The peptide concentration required to achieve a half-maximal IFN-γ response (EC_50_) was determined.

### HLA class I genotyping

Four-digit HLA class I genotyping was performed by direct sequencing methods as described [76]. The data were analyzed and alleles were assigned using Assign-SBT version 3.5 (Conexio Genomics, Applecross, Australia).

### Statistical analyses

Mann-Whitney and Wilcoxon-matched paired tests were performed using GraphPad Prism version 6.00 (San Diego, CA). Analyses of the functional avidity of CD8 T-cell responses were performed on log_10_-transformed data using non-parametric tests. Associations among variables were performed by Spearman test. Rank correlations matrix and linear regression analysis were performed after log_10_ transformation of variables using R software. Bonferroni corrections for multiple analyses were applied. Regarding SPICE analyses of the flow-cytometry data, comparison of distributions was performed using a Student's *t*-test and a partial permutation test as described [75]. Furthermore, mixed-effect linear models were used to assess the evolution of functional avidity as a function of the time and virus rebound, as described [39], [40]. In brief, let Y_ij be the measured avidity for subject i at time j (time_ij) and rebound_ij, the covariate coded as 1 if a patient i is an on-therapy at time j and coded as 0 if not on therapy (off-therapy). We fitted the following mixed effect linear model: Y_ij = (β_0+r_i)+(β_1)time_ij+(β_2)rebound_ij+ε_ij where β_0 is the global mean, β_1 the effect of the time on avidity, β_2 the effect of the virus rebound on avidity, r_i the random effect which represents the individual deviation from the global intercept and ε ij are independent measurement errors with mean zero. The interaction between time_ij and rebound_ij was tested.

## Supporting Information

Figure S1 **Magnitude and qualitative profiles of HIV-specific CD8 T cells during acute and chronic HIV infections.** **A.** Associations between the magnitude and the functional avidity of HIV-specific CD8 T-cell responses from patients with either acute infection (PHI-B), untreated chronic progressive (CP-B) or non-progressive (LTNP) HIV infection. **B.** Representative flow cytometry examples of the functional profile of HIV-specific CD8 T cells from patients with acute (PHI-B-07 B*4402-_AENLWVTVYY_) and chronic progressive (CP-B-1021; A*2601-_EVIPMFSAL_) and non-progressive (LTNP-013; A*0201-_FLGKIWPSYK_) HIV infection on the basis of the expression of IFN-γ, TNF-α, IL-2 and perforin. **C.** Representative flow cytometry profiles show the identification of HIV-specific CD8 T cells using relevant peptide-MHC class I multimer complexes (upper panels) from patients with acute (PHI-B-1037; B*1402-_DRFYKTLRA_) or chronic progressive (CP-B-11; A*0201-_SLYNTVATL_) and non-progressive (LTNP-2081 A*0201-_SLYNTVATL_) and CD27 and CD28 expression on HIV-specific CD8 T cells (bottom panels). **D.** Representative flow cytometry profiles show the identification of HIV-specific CD8 T cells using relevant peptide-MHC class I multimer complexes (left panels) from patients with acute (PHI-B-1037; B*1402-_DRFYKTLRA_) or chronic progressive (CP-B-11; A*0201-_SLYNTVATL_) and non-progressive (LTNP-2081 A*0201-_SLYNTVATL_) HIV infection and 2B4, PD-1 and CD160 expression on HIV-specific CD8 T cells (right panels).
(PPTX)

Figure S2 **Effect of the combination of Cyclosporin A with ART and T-cell responses.** Analysis of the magnitude and of the functional avidity of HIV-specific CD8 T-cell responses in PHI patients treated for one year with either ART alone or ART + Cycosporin A (CsA).
(PPTX)

Figure S3 **TRBV usage and CDR3 size pattern.** Example of TRBV usage and CDR3 size pattern analysis of B*0702-_GPGHKARVL_-specific CD8 T cells in patient #1023 at week 18, 96 and 125. **A.** Profile of BV families obtained by PCR. **B.** CDR3 size profile obtained by genemapper analysis of BV families. TRB nomenclature is according to Wei *et al.* Immunogenetics (1994). The model used to define CDR3 diversity and renewal is based on Miconnet *et al.* J. Immunol. (2011).
(PPTX)

Table S1 Clinical and virological description of the distinct cohorts of HIV-infected patients.
(PPTX)

Table S2 HIV-derived peptide-MHC class I multimer complexes used in this study.
(PPTX)
