# Supplementary material for: Rapid Perturbation in Viremia Levels Drives Increases in Functional Avidity of HIV-specific CD8 T Cells
Source: PLoS Pathog. 2013 Jul 4;9(7):e1003423. doi: 10.1371/journal.ppat.1003423 (PMC3701695; doi:10.1371/journal.ppat.1003423)
Supplement: Figure S1 — Magnitude and qualitative profiles of HIV-specific CD8 T cells during acute and chronic HIV infections. A. Associations between the magnitude and the functional avidity of HIV-specific CD8 T-cell responses from patients with either acute infection (PHI-B), untreated chronic progressive (CP-B) or non-progressive (LTNP) HIV infection. B. Representative flow cytometry examples of the functional profile of HIV-specific CD8 T cells from patients with acute (PHI-B-07 B*4402-AENLWVTVYY) and chronic progressive (CP-B-1021; A*2601-EVIPMFSAL) and non-progressive (LTNP-013; A*0201-FLGKIWPSYK) HIV infection on the basis of the expression of IFN-γ, TNF-α, IL-2 and perforin. C. Representative flow cytometry profiles show the identification of HIV-specific CD8 T cells using relevant peptide-MHC class I multimer complexes (upper panels) from patients with acute (PHI-B-1037; B*1402-DRFYKTLRA) or chronic progressive (CP-B-11; A*0201-SLYNTVATL) and non-progressive (LTNP-2081 A*0201-SLYNTVATL) and CD27 and CD28 expression on HIV-specific CD8 T cells (bottom panels). D. Representative flow cytometry profiles show the identification of HIV-specific CD8 T cells using relevant peptide-MHC class I multimer complexes (left panels) from patients with acute (PHI-B-1037; B*1402-DRFYKTLRA) or chronic progressive (CP-B-11; A*0201-SLYNTVATL) and non-progressive (LTNP-2081 A*0201-SLYNTVATL) HIV infection and 2B4, PD-1 and CD160 expression on HIV-specific CD8 T cells (right panels). (PPTX) [file ppat.1003423.s001.pptx]

## Slide 1
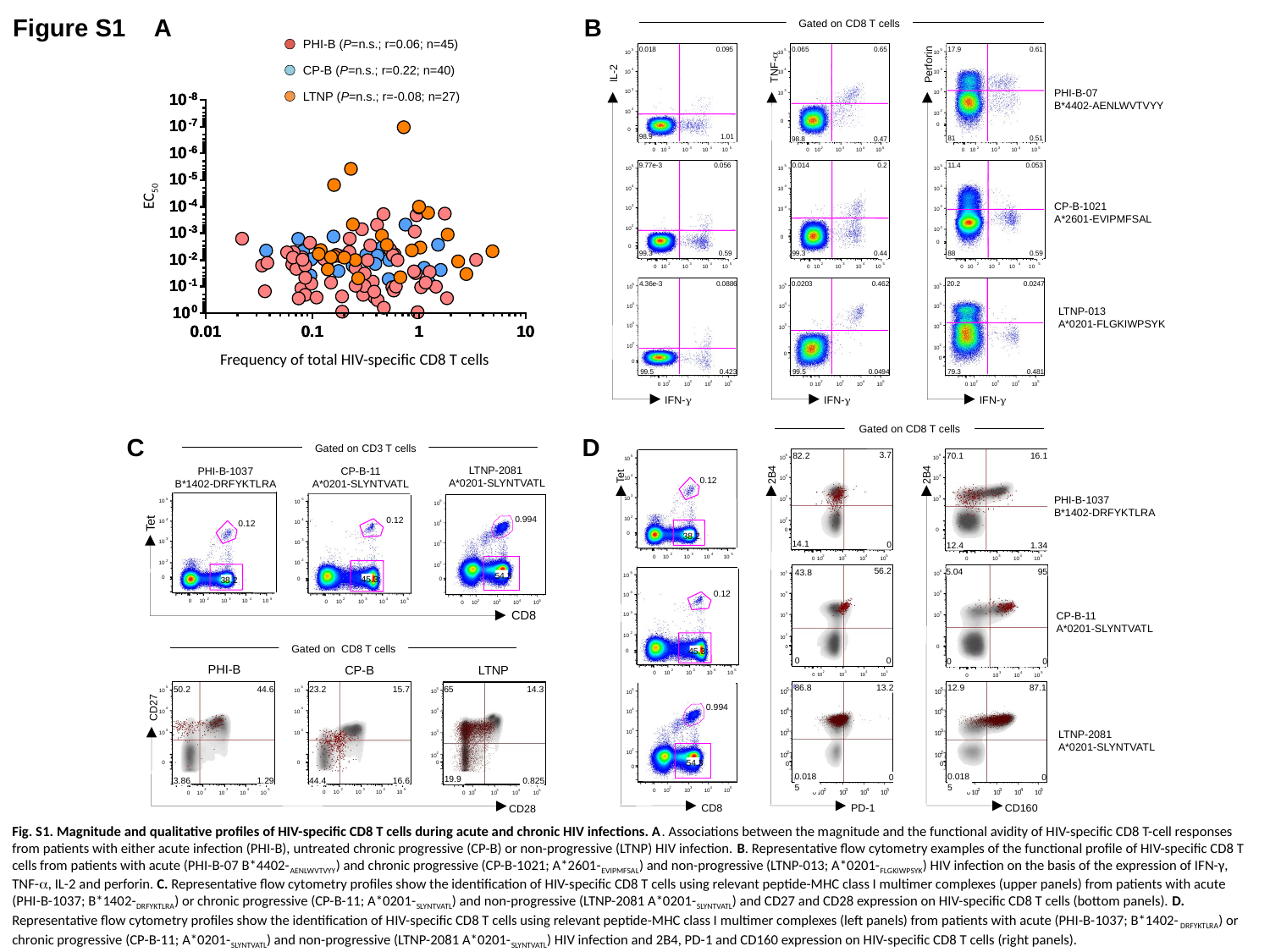

Figure S1
A
B
Gated on CD8 T cells
PHI-B (P=n.s.; r=0.06; n=45)
5
10
4
10
3
10
2
10
0
2
3
4
5
0
10
10
10
10
0.095
0.018
98.9
1.01
5
10
4
10
3
10
0
2
3
4
5
0
10
10
10
10
0.65
0.065
98.8
0.47
5
10
4
10
3
10
2
10
0
2
3
4
5
0
10
10
10
10
0.61
17.9
81
0.51
CP-B (P=n.s.; r=0.22; n=40)
Perforin
TNF-α
IL-2
PHI-B-07
B*4402-AENLWVTVYY
LTNP (P=n.s.; r=-0.08; n=27)
5
10
4
10
3
10
2
10
0
2
3
4
5
0
10
10
10
10
0.056
9.77e-3
99.3
0.59
5
10
4
10
3
10
0
2
3
4
5
0
10
10
10
10
0.2
0.014
99.3
0.44
5
10
4
10
3
10
2
10
0
2
3
4
5
0
10
10
10
10
0.053
11.4
88
0.59
EC50
CP-B-1021
A*2601-EVIPMFSAL
5
10
4
10
3
10
2
10
0
2
3
4
5
0
10
10
10
10
0.0886
4.36e-3
99.5
0.423
5
10
4
10
3
10
0
2
3
4
5
0
10
10
10
10
0.462
0.0203
99.5
0.0494
5
10
4
10
3
10
2
10
0
2
3
4
5
0
10
10
10
10
0.0247
20.2
79.3
0.481
LTNP-013
A*0201-FLGKIWPSYK
Frequency of total HIV-specific CD8 T cells
 IFN-γ
 IFN-γ
 IFN-γ
Gated on CD8 T cells
C
D
Gated on CD3 T cells
LTNP-2081
A*0201-SLYNTVATL
PHI-B-1037
B*1402-DRFYKTLRA
CP-B-11
A*0201-SLYNTVATL
5
10
4
10
3
10
2
10
0
2
3
4
5
0
10
10
10
10
0.12
45.3
5
10
4
10
3
10
2
10
0
2
3
4
5
0
10
10
10
10
0.12
38.2
5
10
4
10
3
10
2
10
0
2
3
4
5
0
10
10
10
10
0.994
54.3
Tet
CD8
Gated on CD8 T cells
PHI-B
CP-B
LTNP
5
10
4
10
3
10
0
2
3
4
5
0
10
10
10
10
5
10
4
10
3
10
0
2
3
4
5
0
10
10
10
10
23.2
15.7
44.4
16.6
5
10
4
10
3
10
2
10
0
2
3
4
5
0
10
10
10
10
14.3
65
19.9
0.825
50.2
44.6
CD27
3.86
1.29
CD28
5
10
4
10
3
10
2
10
0
2
3
4
5
0
10
10
10
10
0.12
38.2
5
10
4
10
3
10
2
10
0
2
3
4
5
0
10
10
10
10
3.7
82.2
14.1
0
5
10
4
10
3
10
0
3
4
5
0
10
10
10
70.1
16.1
12.4
1.34
2B4
2B4
Tet
PHI-B-1037
B*1402-DRFYKTLRA
5
10
4
10
3
10
2
10
0
2
3
4
5
0
10
10
10
10
0.12
45.3
5
10
4
10
3
10
2
10
0
2
3
4
5
0
10
10
10
10
56.2
43.8
0
0
5
10
4
10
3
10
0
3
4
5
0
10
10
10
5.04
95
0
0
CP-B-11
A*0201-SLYNTVATL
5
10
4
10
3
10
2
10
0
2
3
4
5
0
10
10
10
10
5
10
4
10
3
10
2
10
0
2
3
4
5
0
10
10
10
10
86.8
13.2
12.9
87.1
5
10
4
10
3
10
2
10
0
2
3
4
5
0
10
10
10
10
0.994
54.3
LTNP-2081
A*0201-SLYNTVATL
0.0185
0.0185
0
0
CD8
PD-1
CD160
Fig. S1. Magnitude and qualitative profiles of HIV-specific CD8 T cells during acute and chronic HIV infections. A. Associations between the magnitude and the functional avidity of HIV-specific CD8 T-cell responses from patients with either acute infection (PHI-B), untreated chronic progressive (CP-B) or non-progressive (LTNP) HIV infection. B. Representative flow cytometry examples of the functional profile of HIV-specific CD8 T cells from patients with acute (PHI-B-07 B*4402-AENLWVTVYY) and chronic progressive (CP-B-1021; A*2601-EVIPMFSAL) and non-progressive (LTNP-013; A*0201-FLGKIWPSYK) HIV infection on the basis of the expression of IFN-γ, TNF-α, IL-2 and perforin. C. Representative flow cytometry profiles show the identification of HIV-specific CD8 T cells using relevant peptide-MHC class I multimer complexes (upper panels) from patients with acute (PHI-B-1037; B*1402-DRFYKTLRA) or chronic progressive (CP-B-11; A*0201-SLYNTVATL) and non-progressive (LTNP-2081 A*0201-SLYNTVATL) and CD27 and CD28 expression on HIV-specific CD8 T cells (bottom panels). D. Representative flow cytometry profiles show the identification of HIV-specific CD8 T cells using relevant peptide-MHC class I multimer complexes (left panels) from patients with acute (PHI-B-1037; B*1402-DRFYKTLRA) or chronic progressive (CP-B-11; A*0201-SLYNTVATL) and non-progressive (LTNP-2081 A*0201-SLYNTVATL) HIV infection and 2B4, PD-1 and CD160 expression on HIV-specific CD8 T cells (right panels).
