# Supplementary material for: Rapid Perturbation in Viremia Levels Drives Increases in Functional Avidity of HIV-specific CD8 T Cells
Source: PLoS Pathog. 2013 Jul 4;9(7):e1003423. doi: 10.1371/journal.ppat.1003423 (PMC3701695; doi:10.1371/journal.ppat.1003423)
Supplement: Figure S2 — Effect of the combination of Cyclosporin A with ART and T-cell responses. Analysis of the magnitude and of the functional avidity of HIV-specific CD8 T-cell responses in PHI patients treated for one year with either ART alone or ART + Cycosporin A (CsA). (PPTX) [file ppat.1003423.s002.pptx]

## Slide 1
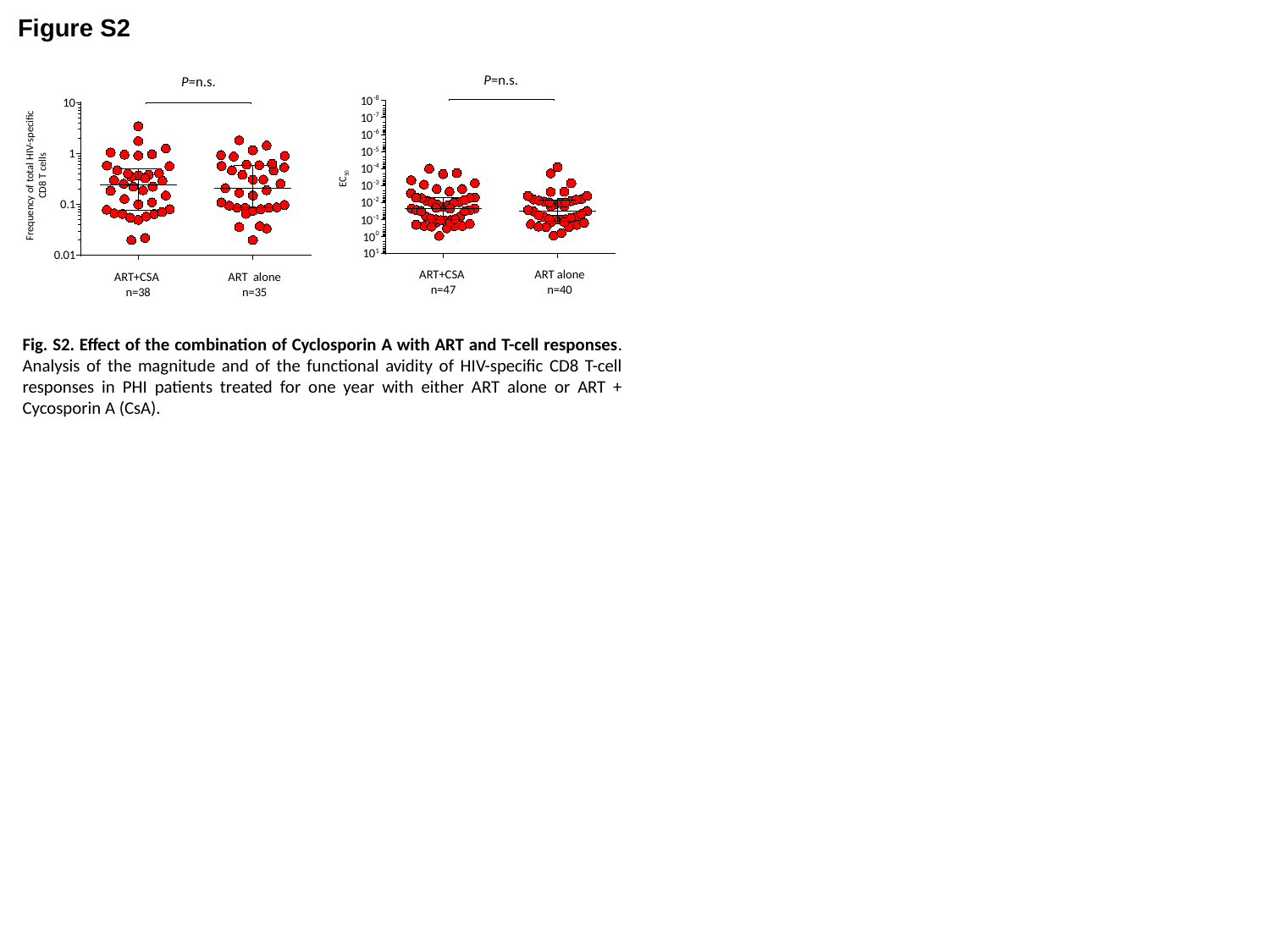

Figure S2
P=n.s.
P=n.s.
10
-8
10
-7
10
-6
10
-5
10
-4
10
-3
10
-2
10
-1
10
0
10
1
10
1
0.1
0.01
Frequency of total HIV-specific CD8 T cells
EC50
ART+CSA
n=47
ART alone
n=40
ART+CSA
n=38
ART alone
n=35
Fig. S2. Effect of the combination of Cyclosporin A with ART and T-cell responses. Analysis of the magnitude and of the functional avidity of HIV-specific CD8 T-cell responses in PHI patients treated for one year with either ART alone or ART + Cycosporin A (CsA).
