# Supplementary material for: Rapid Perturbation in Viremia Levels Drives Increases in Functional Avidity of HIV-specific CD8 T Cells
Source: PLoS Pathog. 2013 Jul 4;9(7):e1003423. doi: 10.1371/journal.ppat.1003423 (PMC3701695; doi:10.1371/journal.ppat.1003423)
Supplement: Figure S3 — TRBV usage and CDR3 size pattern. Example of TRBV usage and CDR3 size pattern analysis of B*0702-GPGHKARVL-specific CD8 T cells in patient #1023 at week 18, 96 and 125. A. Profile of BV families obtained by PCR. B. CDR3 size profile obtained by genemapper analysis of BV families. TRB nomenclature is according to Wei et al. Immunogenetics (1994). The model used to define CDR3 diversity and renewal is based on Miconnet et al. J. Immunol. (2011). (PPTX) [file ppat.1003423.s003.pptx]

## Slide 1
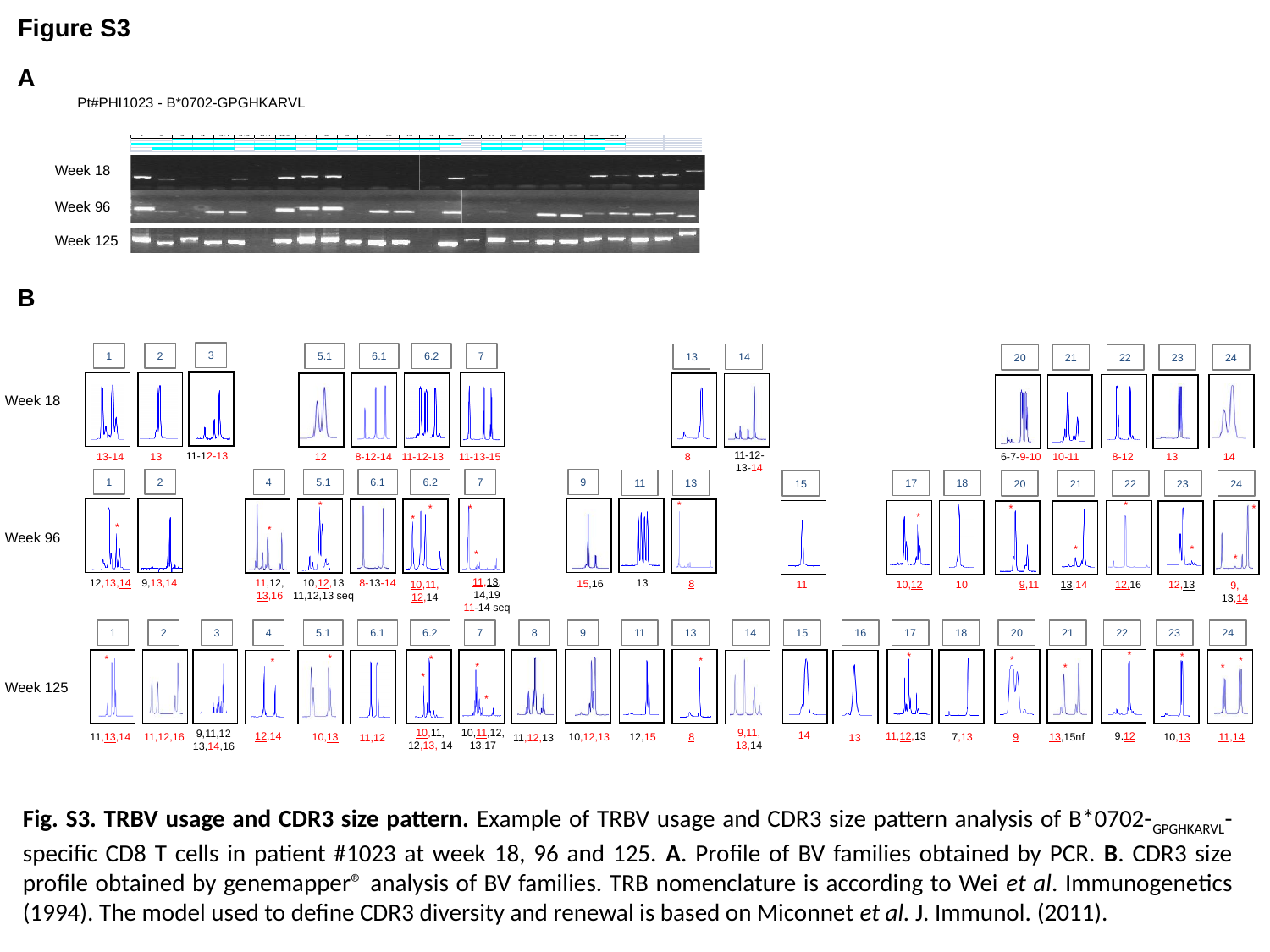

Figure S3
A
Pt#PHI1023 - B*0702-GPGHKARVL
Week 18
Week 96
Week 125
B
3
1
2
5.1
6.1
6.2
7
13
14
20
21
22
23
24
11-12-13-14
11-12-13
13-14
13
12
8-12-14
11-12-13
11-13-15
8
6-7-9-10
10-11
8-12
13
14
1
2
4
5.1
6.1
6.2
7
9
11
13
17
18
15
20
21
22
23
24
*
*
*
*
*
*
*
*
*
*
*
*
*
*
*
11,13,
14,19
11-14 seq
12,13,14
9,13,14
11,12,
13,16
10,12,13
11,12,13 seq
8-13-14
13
10,12
10
9,11
13,14
12,16
8
15,16
11
12,13
10,11,
12,14
9,
13,14
1
2
3
9,11,12
13,14,16
4
5.1
6.1
6.2
7
8
11,12,13
9
11
13
14
9,11,
13,14
15
16
17
18
20
21
22
23
24
*
*
*
*
*
*
*
*
*
*
*
*
*
*
*
10,11,
12,13, 14
10,11,12,
13,17
14
11,12,13
9.12
12,14
8
11,13,14
11,12,16
10,13
10,13
11,14
10,12,13
12,15
7,13
9
13,15nf
11,12
13
Week 18
Week 96
Week 125
Fig. S3. TRBV usage and CDR3 size pattern. Example of TRBV usage and CDR3 size pattern analysis of B*0702-GPGHKARVL-specific CD8 T cells in patient #1023 at week 18, 96 and 125. A. Profile of BV families obtained by PCR. B. CDR3 size profile obtained by genemapper® analysis of BV families. TRB nomenclature is according to Wei et al. Immunogenetics (1994). The model used to define CDR3 diversity and renewal is based on Miconnet et al. J. Immunol. (2011).
