# Supplementary material for: Rapid Perturbation in Viremia Levels Drives Increases in Functional Avidity of HIV-specific CD8 T Cells
Source: PLoS Pathog. 2013 Jul 4;9(7):e1003423. doi: 10.1371/journal.ppat.1003423 (PMC3701695; doi:10.1371/journal.ppat.1003423)
Supplement: Table S1 — Clinical and virological description of the distinct cohorts of HIV-infected patients. (PPTX) [file ppat.1003423.s004.pptx]

## Slide 1
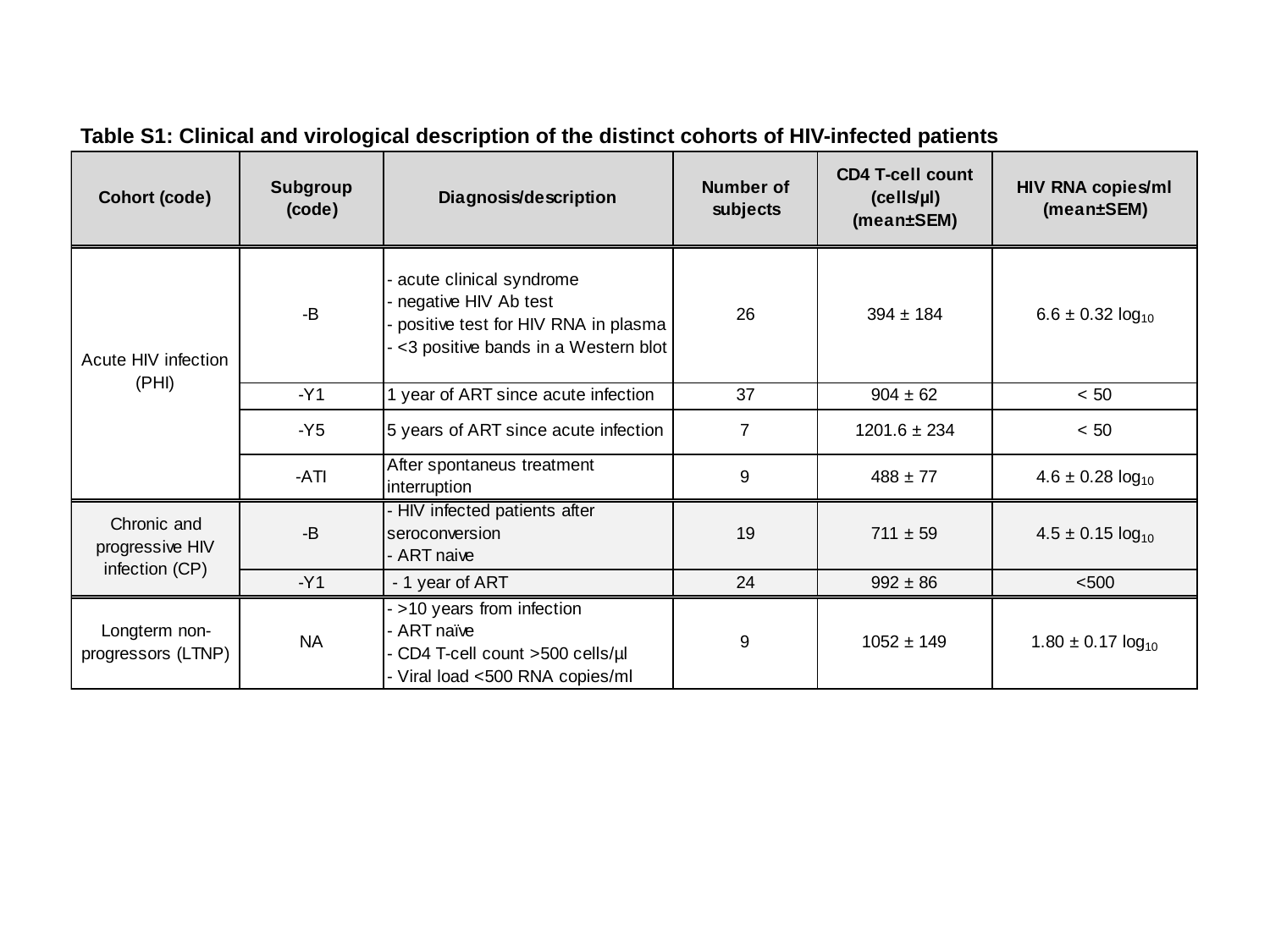

Table S1: Clinical and virological description of the distinct cohorts of HIV-infected patients
