# Supplementary figures and images for: Rapid Perturbation in Viremia Levels Drives Increases in Functional Avidity of HIV-specific CD8 T Cells
Source: PLoS Pathog. 2013 Jul 4;9(7):e1003423. doi: 10.1371/journal.ppat.1003423 (PMC3701695; doi:10.1371/journal.ppat.1003423)

## Slide 1
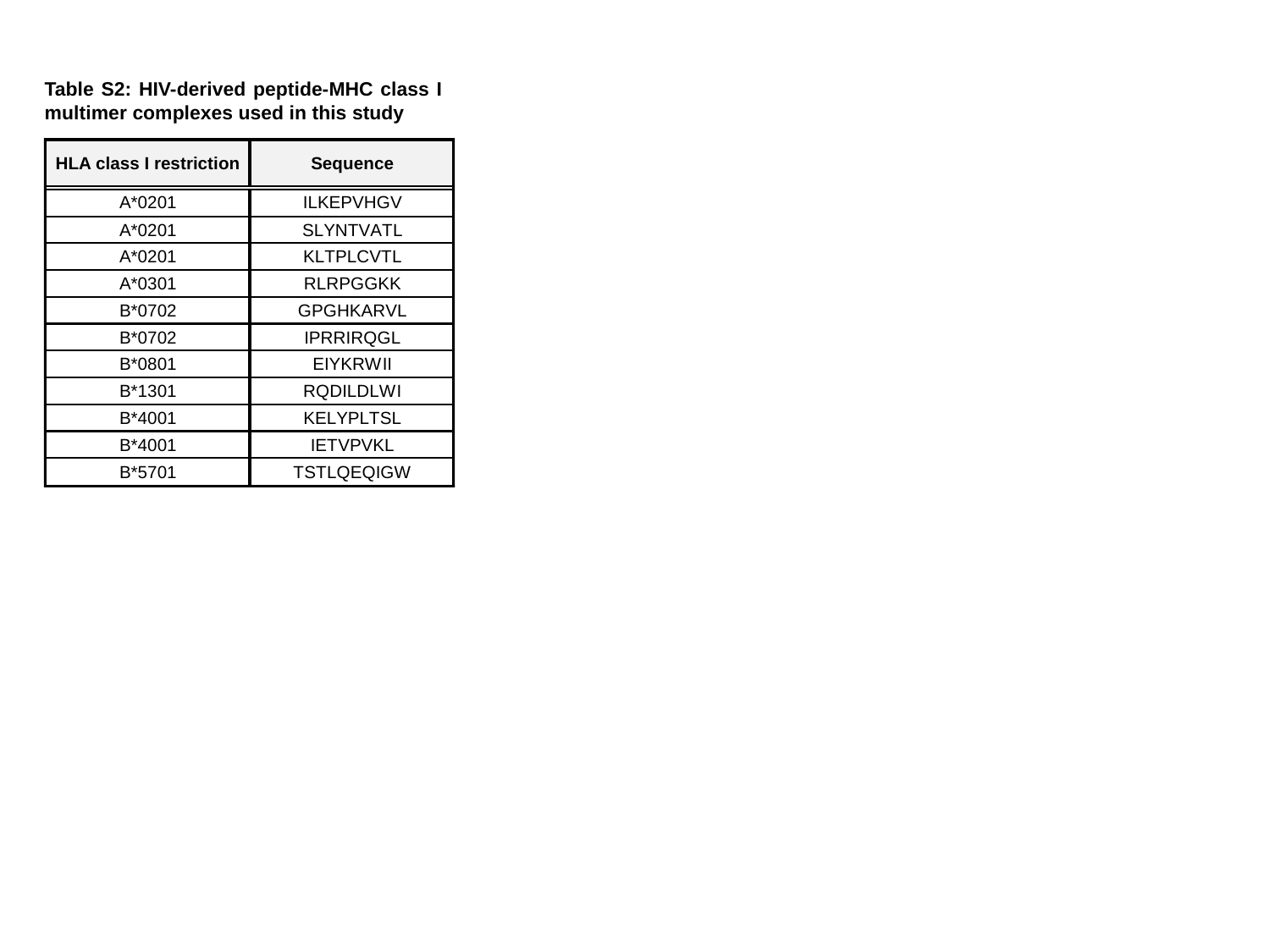

Table S2: HIV-derived peptide-MHC class I multimer complexes used in this study

Supplement: Table S2 — HIV-derived peptide-MHC class I multimer complexes used in this study. (PPTX) [file ppat.1003423.s005.pptx]
